# Costs and Benefits of Chemical Defence in the Red Alga *Bonnemaisonia hamifera*

**DOI:** 10.1371/journal.pone.0061291

**Published:** 2013-04-09

**Authors:** Göran M. Nylund, Swantje Enge, Henrik Pavia

**Affiliations:** Department of Biological and Environmental Sciences - Tjärnö, University of Gothenburg, Strömstad, Sweden; University of New South Wales, Australia

## Abstract

A number of studies have shown that the production of chemical defences is costly in terrestrial vascular plants. However, these studies do not necessarily reflect the costs of defence production in macroalgae, due to structural and functional differences between vascular plants and macroalgae. Using a specific culturing technique, we experimentally manipulated the defence production in the red alga *Bonnemaisonia hamifera* to examine if the defence is costly in terms of growth. Furthermore, we tested if the defence provides fitness benefits by reducing harmful bacterial colonisation of the alga. Costly defences should provide benefits to the producer in order to be maintained in natural populations, but such benefits through protection against harmful bacterial colonisation have rarely been documented in macroalgae. We found that algae with experimentally impaired defence production, but with an externally controlled epibacterial load, grew significantly better than algae with normal defence production. We also found that undefended algae exposed to a natural epibacterial load experienced a substantial reduction in growth and a 6-fold increase in cell bleaching, compared to controls. Thus, this study provides experimental evidence that chemical defence production in macroalgae is costly, but that the cost is outweighed by fitness benefits provided through protection against harmful bacterial colonisation.

## Introduction

Organisms have evolved a variety of defence mechanisms to defend themselves against their natural enemies, and most theories about the evolution of defences assume that these mechanisms are costly [1]–[3]. Conventionally, a cost of defence is defined as a reduction in fitness in the absence of the targeted enemy [4]. Defence costs will determine whether a stable polymorphism of resistant and susceptible genotypes can be maintained in a population [5], and are considered as a significant driving force for the evolution of inducible defences [6].

Plants commonly use chemical defences to protect themselves from attacks by herbivores and pathogens. Although earlier studies provided mixed results [7], there is now growing evidence that chemical defence production generally is costly for terrestrial vascular plants [6], [8]–[11]. However, nonvascular plants like marine macroalgae differ from vascular plants in their structure, tissue organisation and physiology, which may make the magnitude of these costs and their underlying mechanisms different. In comparison to vascular plants, macroalgae have poorly developed internal transport systems for nutrients and photosynthetic products, but instead have the capacity to photosynthesise and absorb nutrients throughout most part of the thallus [12], [13]. Only a few previous studies have focused on the costs of chemical defences in macroalgae and they are almost exclusively restricted to one class of metabolites, the brown algal phlorotannins, where significant costs have been inferred from negative phenotypic correlations between growth and levels of defence metabolites (reviewed by [14]). One notable exception is the work by Dworjanyn et al. [15] on the red alga *Delisea pulchra* and its production of brominated furanones. A significant cost of defence production was detected in *D. pulchra* germlings by culturing the alga with and without access to bromine. Such culturing manipulations offer a powerful tool to examine costs of chemical defences in macroalgae where poor mechanistic knowledge of chemical defence production restricts the use of the chemical elicitation or transgenic methods more commonly used for terrestrial plants [16], [17].

Experimental manipulations of chemical defences by preventing access to bromine are particularly well suited for red algae where bromine in form of bromide is commonly incorporated into secondary metabolites [18], but considered to be non-essential for normal growth and primary metabolism [15], [19]. One interesting candidate for such experiments is the red alga *Bonnemaisonia hamifera* that produces a brominated compound, 1,1,3,3-tetrabromo-2-heptanone, with broad-spectrum biological activity. In recent studies it has been shown that the inhibition of herbivory as well as recruitment of competitors caused by this compound may provide significant fitness benefits to the alga [20], [21]. This should select for the maintenance of the defence in natural populations even if it is costly to produce. The same compound has also been shown to have an ecological role in deterring bacterial colonisation of the algal thallus surface [22], [23], but the fitness benefit from this inhibition is less clear. In general, very little is known about the impacts of natural bacterial colonisation on the fitness of macroalgae and how this contributes to the evolution of algal chemical defences [24], [25], despite that it is well known that macroalgae harbour diverse and abundant populations of bacteria [26]–[30].

We used *B. hamifera* as a model species to examine if chemical defence production in macroalgae is costly and if chemical inhibition of bacterial surface colonisation provides fitness benefits to macroalgae. To experimentally manipulate the expression of the chemical defence we cultured algal individuals with a natural epibacterial community in media with and without access to bromine. To control for bacterial growth on algae incapable of inhibiting bacteria we also added the alga's own defence metabolite, 1,1,3,3-tetrabromo-2-heptanone, externally to the growth media as a “natural” antibiotic. Growth and bleaching (loss of pigments) were compared between control algae and algae with depleted levels of defence metabolites, cultured with and without externally added 1,1,3,3-tetrabromo-2-heptanone. This allowed us to test the specific hypotheses that *i*) the chemical defence production in *B. hamifera* is costly, and *ii*) the chemical defence provides fitness benefits to the alga by inhibiting harmful bacterial colonisation.

## Materials and Methods

### Study organism and collection of algal material

*Bonnemaisonia hamifera* is an introduced species in the North Atlantic Ocean distributed from northern Norway and Iceland to the Canary Islands, and from Labrador to Virginia ([31] and references therein). It has a heteromorphic life cycle, alternating between a diploid tetrasporophyte and a haploid gametophyte. On the Swedish west coast only the tetrasporophytic life-stage is found (i.e. the *Trailiella* phase), without the capacity to form tetrasporangia [31], [32]. It is found at depths of around 0.5 – 15 m, where it grows as small turfs on other algae [33]. *Bonnemaisonia hamifera* grows through apical cell division and its major secondary metabolite, 1,1,3,3-tetrabromo-2-heptanone, is stored in specialised gland cells located between the vegetative cells [22], [34]. The total levels of this compound range from 5 – 50 µg mg^−1^ dry mass (G.M. Nylund & H. Pavia, unpublished) and can be found at the surface of algae at concentrations around 4 µg cm^−2^ in natural populations [22].

Individuals of *B. hamifera* were collected by SCUBA diving in the archipelago outside the Sven Lovén Centre for Marine Sciences – Tjärnö on the Swedish west coast (58°54′N, 11°07′E) and cleared from associated fauna before used in laboratory culture experiments. No protected species were sampled for the study. No specific permits were required for the collection and experimentation of the study organisms, because the centre has a permanent permit issued by the County Administrative Board to collect and study non-protected species within the study area.

### Manipulation of secondary metabolite production and measurement of fitness effects

We experimentally manipulated the production of 1,1,3,3-tetrabromo-2-heptanone by growing specimens of *B. hamifera* in artificial media with and without access to bromine in form of sodium bromide (NaBr) [15]. Two separate experimental setups were used to examine the cost and fitness benefit of the chemical defence. The experimental setup 1 was designed to examine the cost of the defence production. Small algal thalli with natural epibacterial communities were weighed and transferred into 50 ml culture flasks. Seven replicates were cultured in media with and seven replicates without the access to bromine (n = 7) for 26 d with a 16:8 h light:dark cycle. The medium without bromide (Br(-)) was Fries' medium ASP6F [35] with modifications according to [36]. The medium with bromide (Br(+)) was the same except the addition of NaBr to attain a concentration of bromide ions comparable to seawater (96.9 mg l^−1^). The culture media were changed every week. To control for the expected negative effects of epibacteria on algae with impaired production of the defence metabolite, synthesised 1,1,3,3-tetrabromo-2-heptanone [22] dissolved in dimethyl sulfoxide was added into the Br(+) and Br(−) media to give a final concentration of 50 µg ml^−1^ medium (the concentration was decided from pilot studies measuring the health of thalli grown in Br(−) media; data not shown). As it is inorganic bromide that is incorporated into algal secondary metabolites by haloperoxidases [37], [38], the added 1,1,3,3-tetrabromo-2-heptanone should not be readily available as a bromine source for the production of brominated compounds. This method of using the alga's own defence metabolite was much more efficient in maintaining healthy algae during the culture than other methods that were tested to control bacteria, i.e. pretreatment with 1% betadine solution [15], addition of 10 µg ml^−1^ ciprofloxacin to the growth medium [39] or 0.01 mg ml^−1^ penicillin G together with 0.25 mg ml^−1^ germanium dioxide [40], and pretreatment with 0.012% NaOCl followed by incubation in 300 mg l^−1^ ampicillin, 30 mg l^−1^ polymyxin B and 60 mg l^−1^ gentamicin [41] (data not shown). At the end of the experiment, the algal thalli were weighed again to calculate growth as weight change. Growth was considered as a relevant estimator of fitness, because in its northern distribution in the Atlantic *B. hamifera* is mainly spreading through vegetative propagation and rafting [31], [32], which are both enhanced with algal size.

The experimental setup 2 was used to examine the potential fitness benefit provided by the chemical defence when the alga is exposed to natural bacterial surface colonisation and was similar to above, except that no 1,1,3,3-tetrabromo-2-heptanone was added to the media. In addition to growth, the proportion of bleached cells was assessed as a measurement of fitness. Bleaching of cells is a proxy for tissue necrosis and also indicates bacterial infection [24], [25], [42]. Using a light microscope at 100 × magnification, 15 photographs of different parts (randomly chosen) of each replicate thalli (n = 7 for each of the four treatments) were taken and thereafter healthy and bleached cells were counted by visual inspection of the photographs. The prevalence of bleaching was also assessed in the cost experiment in order to check for a possible negative impact of culturing *B. hamifera* without bromine. At the end of the experiment, samples for chemical extractions and quantification of surface associated bacteria were also taken (see below).

### Quantification of 1,1,3,3-tetrabromo-2-heptanone

Algal samples (9.7 mg ± 0.7 mg (SE) tissue carefully dried with precision wipes) were extracted in 2 ml dichloromethane at room temperature overnight. The resulting extract was filtered and analysed using gas chromatography-mass spectrometry (GC-MS) with naphthalene (1 µg ml^−1^) as internal standard according to Nylund et al. [22]. Solutions of synthetic 1,1,3,3-tetrabromo-2-heptanone were used as quantification standard.

### Epibacterial abundance

Epibacterial abundances were quantified by direct counts of stained bacteria on the algal surfaces. For this purpose algal individuals were incubated for 8 min in a solution of 4′6-diamidino-2- phenylindole (DAPI) (0.4 µg ml^−1^) and thereafter bacteria were counted by epifluorescence microscopy (Olympus BX 51 microscope, fluorescence mirror unit U-MNUA2) at a magnification of 1000 × in 20 randomly chosen unit fields (10^−4^ mm^2^ per unit field) [29], [43].

### Statistical analysis

Data from the two experimental setups were analysed separately. The homogeneity of variances for all the obtained data were tested with Cochran's test and data were transformed when required, before further statistical analyses were conducted [44]. Two-tailed t-tests were used to test for differences in 1,1,3,3-tetrabromo-2-heptanone levels, growth, bleaching, and bacterial abundance between the treatments [44].

## Results

### Tissue concentration of 1,1,3,3-tetrabromo-2-heptanone

**Figure 1:**
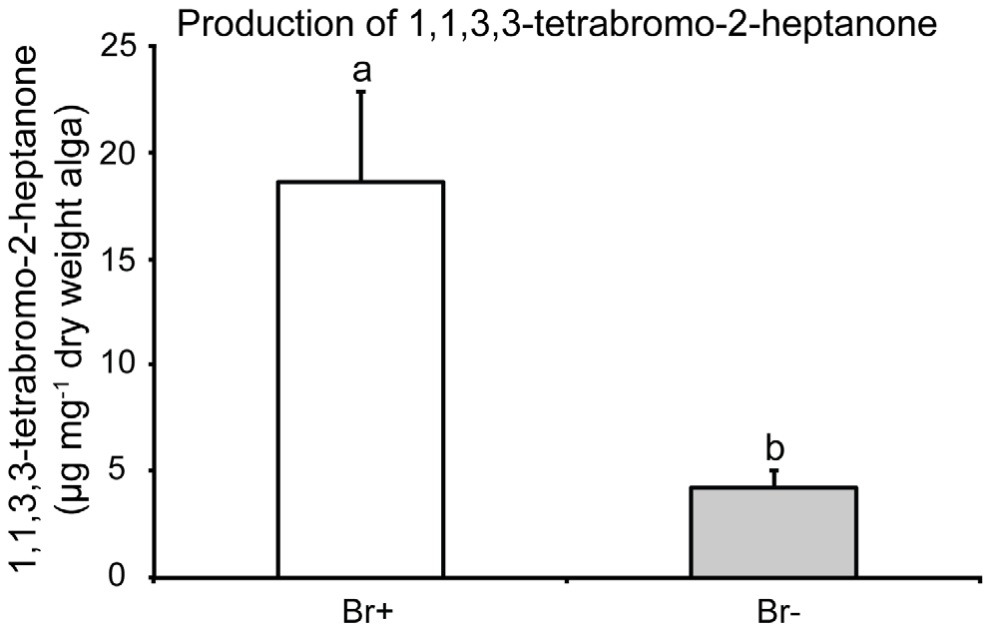
Levels of 1,1,3,3-tetrabromo-2-heptanone in cultured *Bonnemaisonia hamifera*. Algal specimens were cultured for 26 d in bromine (Br+) and bromine free (Br−) media. Means + SE are shown (n = 7). Significant differences are indicated by a, b (t-test).

The omission of bromide from the culture medium significantly decreased the concentration of 1,1,3,3-tetrabromo-2-heptanone in *B. hamifera* (t-test using log-transformed data, t = 5.28, p = 0.00019, Fig. 1). Algae cultured with bromide continued to produce the metabolite at levels comparable to those in algae from natural populations (G.M. Nylund & H. Pavia unpublished).

### Growth and bleaching

**Figure 2:**
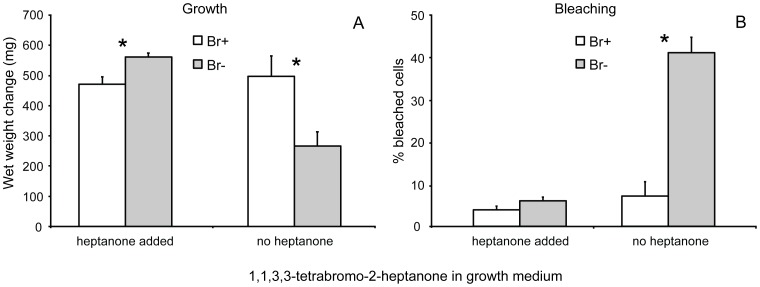
Growth (A) and bleaching (B) of cultured *Bonnemaisonia hamifera*. Algal specimens were cultured for 26 d in bromine (Br+) and bromine free (Br−) media with and without addition of 1,1,3,3-tetrabromo-2-heptanone. Means + SE are shown (n = 7). Asterisks indicate significant differences between treatments (t-tests).

Algal individuals cultured with external addition of 1,1,3,3-tetrabromo-2-heptanone grew significantly more in Br(−) medium than individuals in Br(+) medium (t-test, *t* = 3.22, p = 0.0074, Fig. 2A). When cultured without external addition of 1,1,3,3-tetrabromo-2-heptanone, algae in Br(−) medium grew significantly less (t-test, *t* = 2.83, p = 0.015, Fig. 2A) and had significantly higher proportion of bleached cells (t-test, *t* = 6.95, p = 0.00044, Fig. 2B) than individuals in Br(+) medium. No difference in bleaching between algae cultured in Br(−) and Br(+) medium was detected when 1,1,3,3-tetrabromo-2-heptanone was externally added to the growth medium (t-test, t = 1.58, p = 0.14, Fig. 2B).

### Abundance of surface associated bacteria

**Figure 3:**
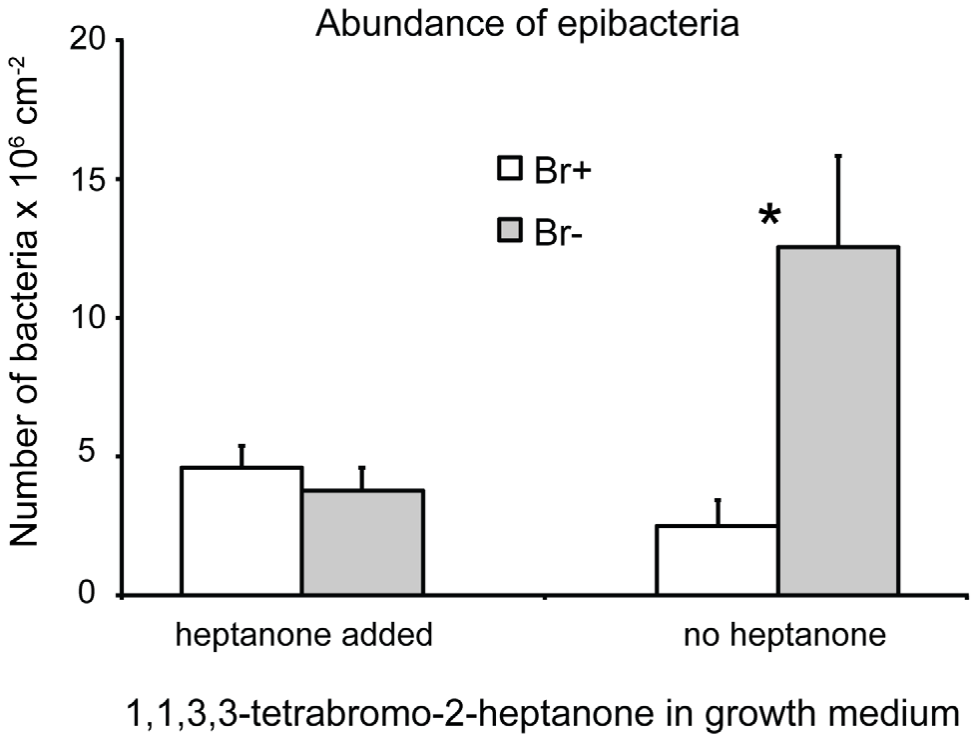
Abundance of surface associated bacteria on cultured *Bonnemaisonia hamifera*. Algal specimens were cultured for 26 d in bromine (Br+) and bromine free (Br−) media with and without addition of 1,1,3,3-tetrabromo-2-heptanone. Means + SE are shown (n = 7). Asterisks indicate significant differences between treatments (t-tests).

Algal individuals cultured without external addition of 1,1,3,3-tetrabromo-2-heptanone had significantly more bacteria on their surface in Br(−) compared to Br(+) medium (t-test using square root transformed data, *t* = 3.68, p = 0.0032, Fig. 3). No difference in bacterial abundance between algae from Br(−) and Br(+) treatments was detected when external addition of 1,1,3,3-tetrabromo-2-heptanone was used in the growth media (t-test, *t* = 0.74, p = 0.48, Fig. 3).

## Discussion

We found a significantly higher growth for individuals of *B. hamifera* with impaired production of defence metabolites, when bacterial abundance was externally controlled. After four weeks in culture where bacterial growth was inhibited by external addition of 1,1,3,3-tetrabromo-2-heptanone, algae with impaired defence metabolite production had grown on average 20% more than algae with normal metabolite production. This is consistent with our hypothesis that the chemical defence of *B. hamifera* is costly. We also found that algae with impaired defence metabolite production were unable to control its epibacterial load. At the end of the experiment in which no 1,1,3,3-tetrabromo-2-heptanone was added to the media, individuals cultured without bromide suffered from remarkably high bleaching and grew significantly less compared to the controls. This is consistent with our second hypothesis that the chemical defence provides fitness benefits by protecting against harmful bacterial surface colonisation.

A few previous studies have also indicated that chemical defences can be costly in macroalgae, but except for the study by Dworjanyn et al. [15] these studies have focused on one class of metabolites, the brown algal phlorotannins [14]. Furthermore, these studies almost exclusively inferred the costs from negative phenotypic correlations between growth and levels of defence metabolites. Conclusions based on phenotypic correlation studies must be viewed with caution because environmental covariances may cause the phenotypic covariances to differ in both sign and magnitude from the underlying genotypic covariances [45], [46]. In contrast, our manipulative approach to impair the production of 1,1,3,3-tetrabromo-2-heptanone offered a more direct test of the costs of defence production, and thus provides strong experimental evidence that chemical defences in macroalgae can be costly. Studies on terrestrial plants have used other manipulative tools to test for the costs of defences, i.e. specific elicitors of inducible chemical defence production, or mutants and transgenic plants. For example, Baldwin [16] found decreased seed production in *Nicotiana attenuata* plants that had been elicited with methyl ester of jasmonic acid (MeJA) to induce increased levels of nicotine. Heidel et al. [47] found that mutants of *Arabidopsis thaliana* with a constitutive activation of the systemic acquired resistance (SAR) signalling pathway generally had lower fitness compared to the wild type. In another study on *N*. *attenuata*, Zavala et al. [17] showed that transgenic plants with either low or no production of trypsin proteinase inhibitors grew faster and taller, flowered earlier, and produced more seed capsules.

The results from studies on terrestrial vascular plants may, however, not necessarily reflect the costs of defence production in macroalgae. Macroalgae are structurally less complex with more limited translocation between cells, but with the capacity to absorb nutrients and have photosynthetic activity throughout the thallus [12], [13]. As pointed out by Dworjanyn et al. [15], this implies that a trade off between growth and defence production is probably not to be expected for most of the cells (except the meristem). This should further suggest that the costs of defences will vary among species according to the specific ratio between meristematic and non-meristematic cells, as well as during the lifetime of a species when this ratio is higher in the less differentiated stages. This reasoning may explain why we found significant costs of chemical defence production for macroscopic *B. hamifera* thalli, while costs in terms of growth were only detected for germlings, but not for larger individuals, of the red alga *Delisea pulchra* [15]. The ratio between meristematic and non-meristematic cells in adults is considerably lower in *D. pulchra* compared to *B. hamifera*.

The total cost for the production of 1,1,3,3-tetrabromo-2-heptanone is probably higher than observed in this study, because the culturing of *B. hamifera* without access to bromine does not inhibit the production of gland cells (Nylund GM, unpublished; see also [48], [49]). The gland cells are used for storage of the defence metabolites [22], [34] and the production and maintenance of these cells should require allocation of both energy and nutrients [6]. Algae cultured in the Br(−) medium without the addition of 1,1,3,3-tetrabromo-2-heptanone contained low levels of the defence compound at the end of the experiment, but this was most likely residuals from the tissue used to start the culture rather than a result of an on-going production. Regardless, the total allocation cost of producing 1,1,3,3-tetrabromo-2-heptanone represents a selective disadvantage to defended genotypes in enemy-free environments (e.g. the absence of herbivores, epiphytic microbes or algal competitors), and the defence should thus be selected against unless it provides significant fitness benefits in natural populations.

We found that the production of 1,1,3,3-tetrabromo-2-heptanone provides *B. hamifera* with significant fitness benefits through protection against harmful bacterial colonisation. After four weeks in culture, individuals with impaired production of defence metabolites had five times more bacteria on their surface, and experienced a substantial reduction in growth as well as a nearly 6-fold increase in bleaching, compared to the controls. Increased development of an epibiotic microbial film may have affected *B. hamifera* negatively by decreasing the uptake of nutrients from the surrounding water and/or by reducing the exudation of toxic waste products from the alga [50]. Alternatively, the impairment of defence production may have increased the growth of pathogenic bacteria [25]. Ideally, studies on the effects of bacteria should be done in the field where algae will be exposed to a natural assemblage of bacteria. However, by starting the culture with algae having a natural community of epibacteria it is likely that the negative effects we observed at the end of the experiment reflected the impacts of bacteria on undefended genotypes in natural populations. Only two studies have previously shown that algal chemical defences can provide fitness benefits through protection against harmful bacterial colonisation. Both studies focused on the red alga *Delisia pulchra* and showed that its chemical defence inhibits bacterially caused bleaching in the field as well as bleaching caused by *Nautella* sp. R11 (former *Ruegeria* sp. R11), a naturally occurring pathogen on *D. pulchra*, in the laboratory [24], [25]. These studies, together with the results of our study, suggest that bacteria constitute a strong selective agent for the evolution of chemical defences in marine macroalgae. It should be noted, however, that bacteria can also be beneficial for marine macroalgae. For example, specific bacteria are necessary for maintaining the common morphology of foliose green algae (Ulvaceae and Monostromaceae) [51] and bacteria living on the surface of algae can provide microbial defence against fouling organisms [52]. It is likely that some bacteria are essential also for *B. hamifera*, because the algae rapidly became senescent when we used commercial broad-spectrum antibiotics in the culture media. The alga's own defence metabolite, on the other hand, maintained healthy algae when added to the media, possibly by selecting for essential bacteria while deterring pathogens.

In conclusion, this study shows that the chemical defence production in *B. hamifera* is costly, but provides significant fitness benefits by protecting against harmful bacterial colonisation. The benefits of the defence in response to other threats, i.e. herbivory and recruitment of competitors [20], [21], should further outweigh the costs of the defence in this alga. The use of defence metabolites with multiple functions should be an effective strategy to increase the net benefits of the production of costly defences.
